# Situs inversus totalis with local metastasis of gallbladder carcinoma and variation of the common hepatic artery

**DOI:** 10.1186/s12876-022-02377-9

**Published:** 2022-07-26

**Authors:** Cheng Zhang, Bo Zhang, Haifeng Huang, Qida Hu, Yibing Jin, Qingsong Yu, Junsen Wang, Xin Zhang, Yun Zhang

**Affiliations:** 1 Department of Hepatobiliary and Pancreatic Surgery, Zhejiang Provincial Key Laboratory of Pancreatic Disease, The First Affiliated Hospital, Zhejiang University School of Medicine, Hangzhou, 310003 China; 2 Department of Hepatobiliary and Pancreatic Surgery, Shengzhou Branch Hospital of the First Affiliated Hospital, Zhejiang University School of Medicine, Shengzhou, 312400 China; 3 Department of Pathology, Shengzhou Branch Hospital of the First Affiliated Hospital, Zhejiang University School of Medicine, Shengzhou, 312400 China; 4 Department of Radiology, The First Affiliated Hospital, Zhejiang University School of Medicine, Hangzhou, 310003 China; 5 Department of Nutrition, The First Affiliated Hospital, Zhejiang University School of Medicine, Hangzhou, 310003 China

**Keywords:** Anatomic variation, Case reports, Gallbladder neoplasms, Neoplasm metastasis, Situs inversus

## Abstract

**Background:**

Situs inversus totalis (SIT) is a rare congenital anomaly characterized by a complete transposition of all the viscera. SIT cases were usually reported because of the presence of tumors, leading to false association between them. Therefore, any research that advances our understanding on SIT is highly required. This study firstly describes a very rare case of SIT with “jumping” metastasis to pancreas of gallbladder carcinoma.

**Case presentation:**

A 69-year-old female patient presented at our hospital with complaints of one month of epigastric pain was studied. She had not sought for treatment prior the visit. Imaging examinations of this patient revealed SIT and a variation of the common hepatic artery with concomitant tumors of gallbladder and pancreas. However, there was no evidence of distant metastases beyond the abdominal cavity. She underwent a combination of radical cholecystectomy, total pancreatectomy, splenectomy and hepatic artery-splenic artery reconstruction. Histological analyses revealed metastasis of the gallbladder carcinoma in to the pancreas. Although the patient opted against chemotherapy, she survived without tumor for 16 months following the surgery. A review of the current literature on association with SIT and tumor occurrence was presented.

**Conclusions:**

It is a great surgical challenge for the resection of multicenter hepatobiliary and pancreatic tumors in such rare SIT anatomical abnormalities with vascular variants. A reliable surgical plan based on detailed preoperative imaging and intraoperative anatomical exploration is crucial to achieving radical resection.

## Background

Situs inversus totalis (SIT) is an abnormal anatomy of the visceral organs characterized by organ transposition or complete inversion of the thoracic and abdominal organs. In the general population, the incidence of SIT ranges from 1/8000 to 1/25,000 [1]. Although SIT with underlying digestive system tumor has been reported, there is no evidence SIT and tumorigenesis are linked [2]. Because of the frequent malformations of transposed organs as well as vascular and nervous anatomical variations that constraint surgical resection, special attention should be paid to the diagnosis and preoperative staging [1, 3, 4].

Precise and careful imaging diagnosis before surgery can not only avoid misdiagnosis and missed diagnosis, but also establish confidence for R0 radical surgery. This study reports for the first time, a very rare case of SIT accompanied with metastatic gallbladder carcinoma and hepatic artery type IX variation.

## Case presentation

**Fig. 1:**
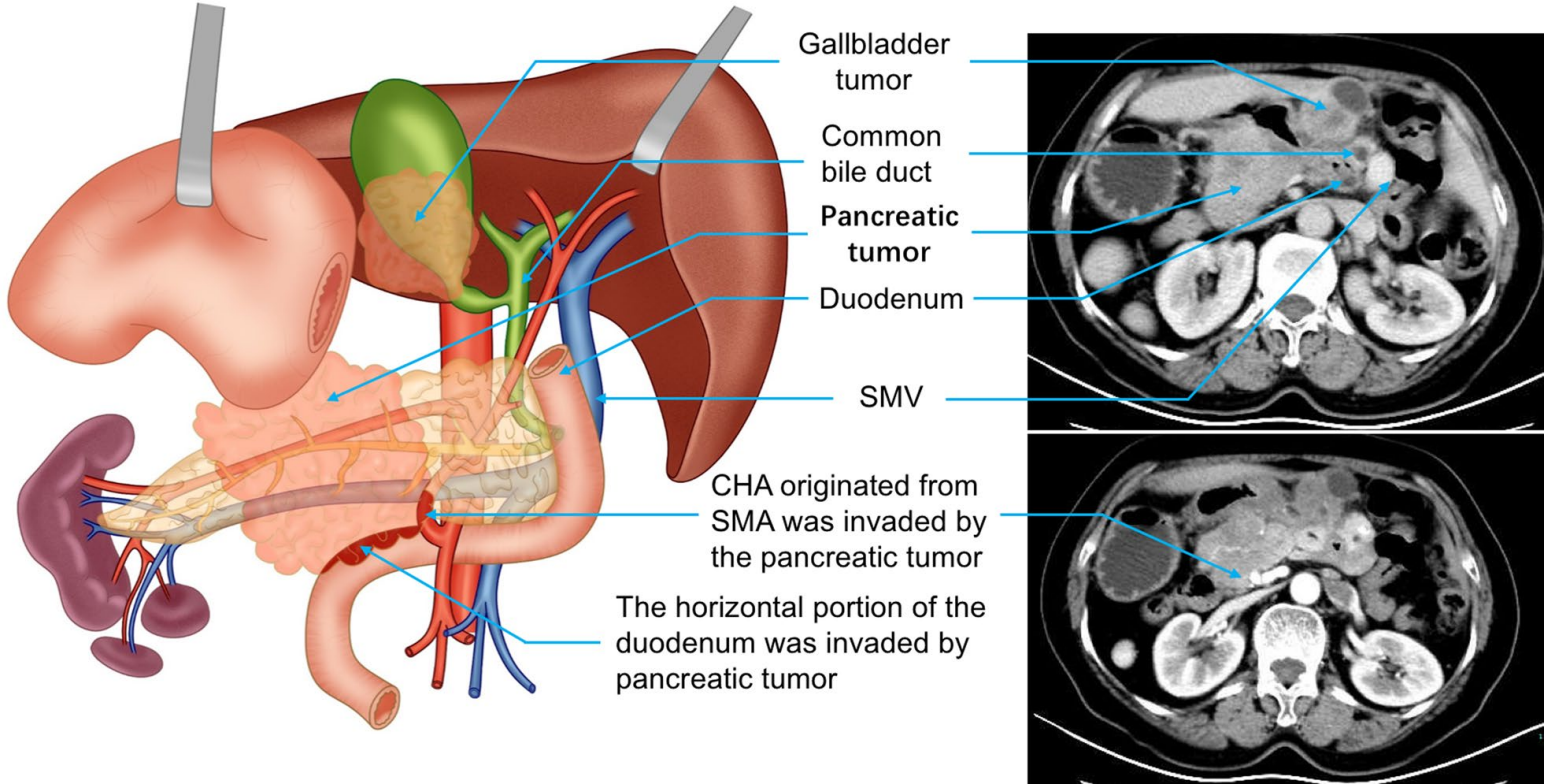
The representative preoperative non-enhanced and enhanced CT investigation of the patient with SIT and gallbladder tumor and pancreatic tail tumor and schematic diagram for the anatomical interpretation of the patient

A 69-year-old female was admitted at our hospital after complaining for epigastric pain for one month. She had not received any treatment regarding the complication prior to the visit. She had no nausea and vomiting, no diarrhea, and no cachexia. Physical examination revealed mild tenderness in the right upper abdomen, but no rebound pain, no jaundice, no abdominal mass and other signs were normal. Blood tests showed normal tumor markers such as alpha fetoprotein, carcinoembryonic antigen, CA125, CA199, CA153, except CA724 which was 7.61 U/ml (reference value 0.00–6.90 U/ml). There were no significant abnormalities in liver function, kidney function, blood routine, urine routine, stool routine, and coagulation function. Radiological examinations revealed SIT that included symmetrical reversal of the liver, stomach, pancreas, duodenum, gallbladder, biliary tract and spleen. In addition, the entire colon was located in the left abdominal cavity and iliac fossa. However, the portal vein which was normal. Other complications included hepatic artery type IX variation, the common hepatic artery (CHA) arising from the superior mesenteric artery (SMA). She additionally presented with gallbladder and pancreatic tail tumors and polysplenia. There was no evidence of metastasis to distant organs beyond the abdominal cavity (Fig. 1). The two sons, a grandson, granddaughter, younger brother, two older sisters, three younger sisters and 11 nephews to the woman underwent computerized tomography (CT) screening. Except for one son with multiple spleens, the rest of the relatives displayed normal organ anatomy.

**Fig. 2:**
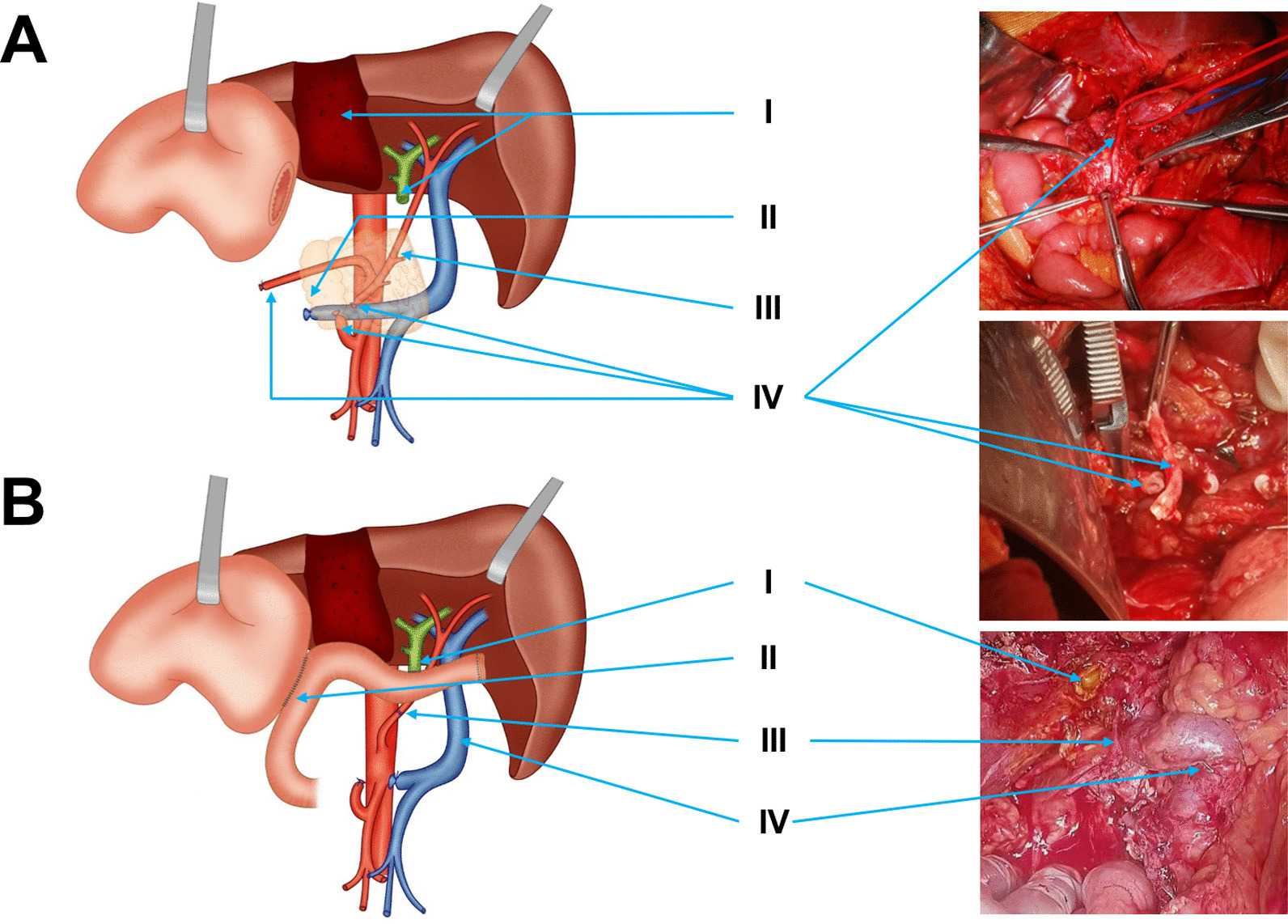
Operation process (**A**) and results (**B**) of the patient. **A** I. Gallbladder mass + partial hepatectomy, common bile duct disconnection. II. Resection of body and tail of pancreas and mass + polysplenectomy. III. Resection of the pancreatic head. IV. Resection of CHA from SMA. **B** I. Cholangiojejunostomy. II. Gastrojejunostomy. III. Anastomization of the proximal end of splenic artery and the distal end of CHA. IV. SMV without pancreatic head support

After careful assessment, the patient underwent a surgery. The whole gallbladder including the “gallbladder bed” was removed, then the common hepatic duct invaded by the gallbladder tumor was cut off (Fig. 2A-I). After the proximal gastric body was cut off, it was discovered that the hepatic artery arose from the SMA, then the pancreatic neck and splenic vein were removed (Fig. 2A-II) and it was found that pancreatic mass had spread to the horizontal segment of the duodenum. SMA branched at the horizontal segment of the duodenum, where one branch entered the mass of the body, whereas there was metastasis to the other branch (CHA) before its passage through the head of the pancreas. Therefore, a decision was made to retain the part of CHA above the pancreas and in the parenchyma of the pancreatic head and the superior mesenteric vein (SMV), but the pancreatic head and the duodenum were resected (Fig.2A-III, IV). Because of the resection of the body and tail of pancreas, the splenic artery freed from the celiac trunk was severed for about 3 cm. The body and tail of the pancreas (including the tumor), multiple spleens and the horizontal segment of the duodenum where the cancer had spread were all removed. Then the end-to-end rebuilding of the splenic and hepatic arteries was performed (Fig. 2B-III), and anastomosis of jejunal-biliary duct and jejunal-stomach were executed to reconstruct the digestive tract (Fig. 2B-I, II). Overall, the surgery involved radical cholecystectomy, total pancreatectomy, splenectomy, cholangiojejunostomy, gastrojejunostomy and proper hepatic artery-splenic artery reconstruction.

**Fig. 3:**
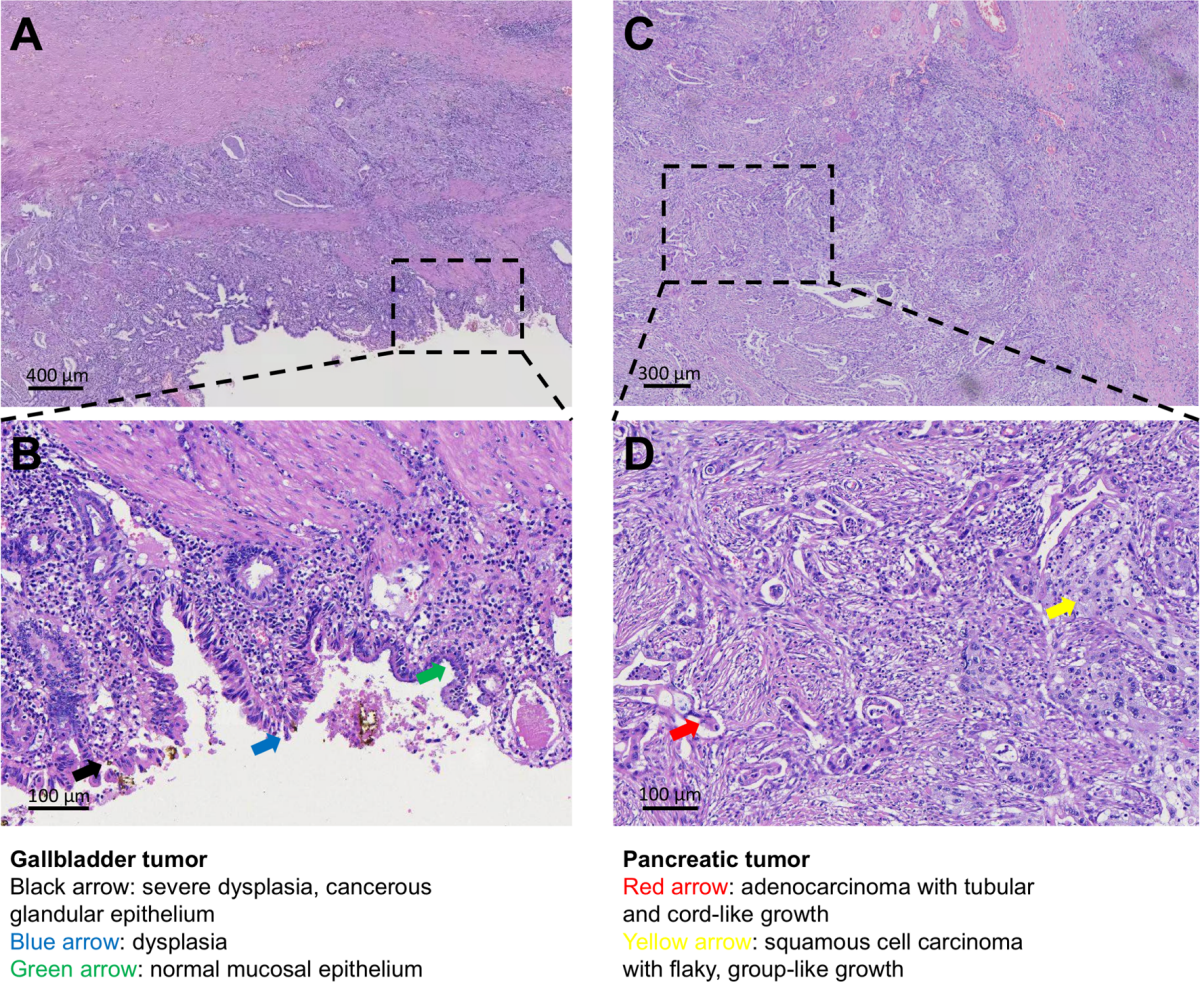
Histological analyses of the gallbladder tumor (**A**, **B**) and pancreatic tumor (**C**, **D**)

**Fig. 4:**
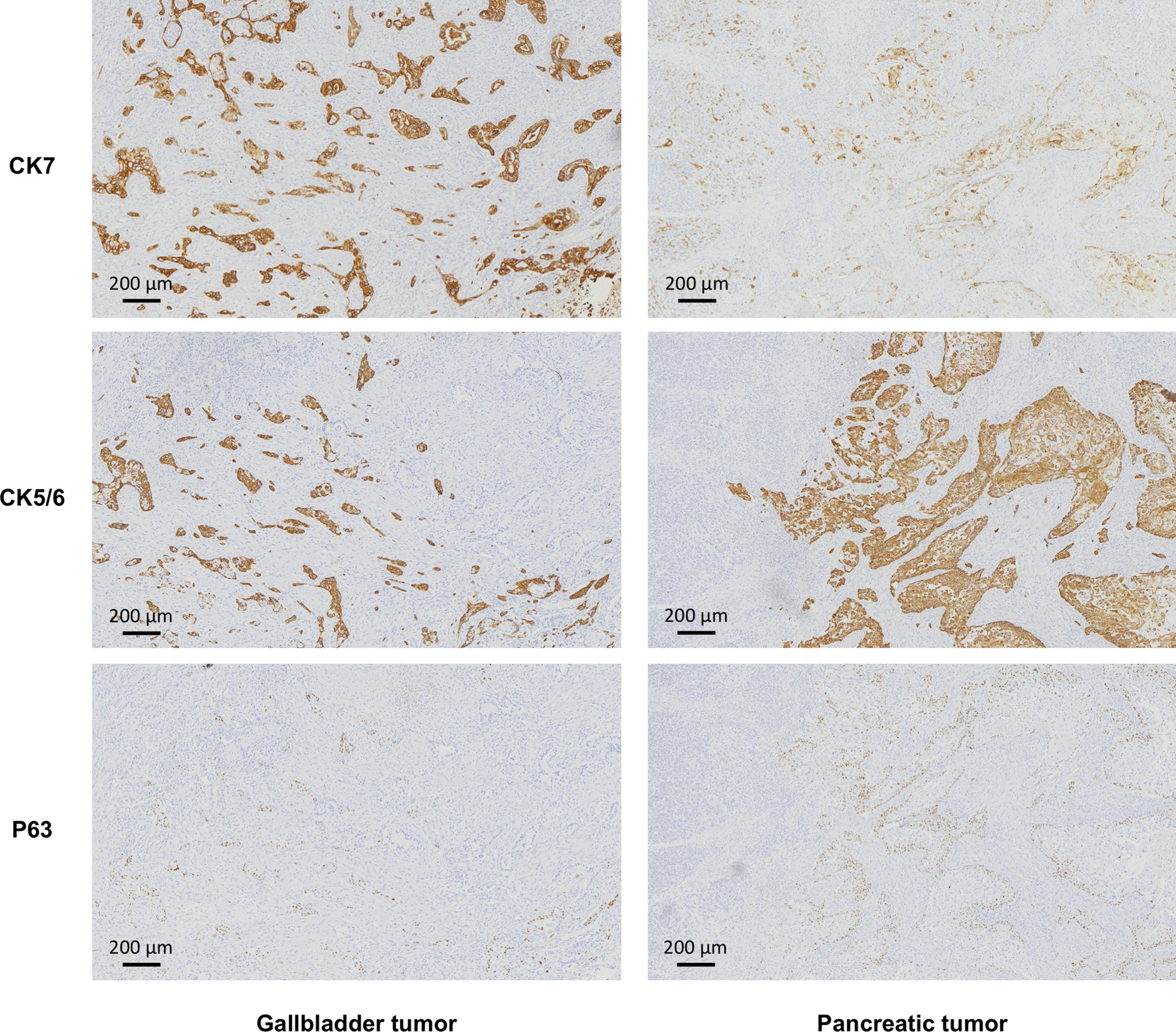
Representative immunohistochemistry results of gallbladder tumor (left) and pancreatic tumor (right)

The adenosquamous carcinoma of the neck and body of the gallbladder measured 3 * 2.5 * 2.5 cm. The cancer had also spread to the liver, pancreas (tumor size 5*5*4 cm), the gastric wall and the muscular layer of the duodenum (horizontal section). Dysplasia was also observed in gallbladder mucosa, which had transformed to adenosquamous carcinoma (Fig. 3A, B). Therefore, gallbladder cancer and pancreatic metastasis (Fig. 3C, D) were the main the main pathological diagnoses. The cancer had not spread to the lining of the common bile duct, stomach, jejunum, posterior peritoneum and the spleen but it had however spread to peripheral lymph nodes (1/5). Overall, the tumor could be described as T3N1M1 according to the AJCC/TNM staging system [5]. Immunohistochemistry tests revealed the same several parameters about the gallbladder and pancreatic tumors (Fig. 4): MUC1(+) MUC2(−) MUC5AC(part +) MUC6(−) CK14(part+) P63(part+) CK5/6(part+) CK20(part+) GATA3(Part+) CDX2(−) Ki67(+ 40%) CK7(+) P53(+), which suggested that the pancreatic tumor metastasized from the gallbladder when combined with the pathological results of HE (Fig. 3), but not a synchronous double cancer [6]. All pathological images with high-resolution were obtained by a Leica DM2500 LED microscope (Leica Microsystems) and captured by Leica Application Suite software (Leica Microsystems), and without any downstream processing.

However, this patient opted against chemotherapy. Blood flow in CHA resumed after reconstruction. The patient did not develop complications such as biliary fistula, intestinal leakage and abdominal infection, and developed no adverse condition after insulin and pancreatin supplement when total pancreatectomy. The tumor had not recurred after 16 months of follow-up.

## Discussion and conclusions

There is no clear consensus on mechanism underlying the development of SIT. Recent reports suggest that SIT results from mutations in the CCDC11 and DNAH11 genes [7, 8]. To date, there is no evidence that SIT exhibit family genetic predisposition. CT scans for 22 close relatives of the patient in this case report revealed no signs of SIT. And SIT cases were usually reported because of the presence of tumors, a phenomenon that creates false impression that SIT patients are more likely to have underlying tumor. Therefore, in-depth researches are needed to unravel the possible relationship between SIT and tumors.

To our knowledge, we firstly described the detailed surgical procedure and techniques in the present case. Although tumors in SIT patients usually require surgical correction, the organ transposition presents a unique challenge. Recent reports showed that hepatobiliary and pancreatic malignancies was occurred in patients with SIT (Table 1) [9–12]. They all suggested that comprehensive preoperative imaging studies and diagnosis are necessary to perform the surgical operation safely, but neither the decision-making nor the surgical procedures were such detailed as this report.

**Table 1:** Recently reported cases of hepatobiliary and pancreatic malignancies in patients with SIT

Year	Age	Sex	Malignancy	Characteristics	Main operation	Stage	Family history	Follow
Present	69	F	Gallbladder carcinoma	“Jumping” local metastasis and the CHA arose from the SMA	Pancreaticoduodenectomy and the CHA reconstruction	T3N1M1	22 close relatives revealed no signs of SIT or tumor	16 months
2019 [9]	62	F	Adenocarcinoma of the duodenal papilla	None	Pancreaticoduodenectomy	T2N1M0	NA	3 months
2018 [2]	56	M	Pancreatic head ductal adenocarcinoma	The CHA arose from the SMA	Pancreaticoduodenectomy and the CHA reconstruction	T3N1M0	NA	12 months
2015 [10]	62	M	Hepatocellular carcinoma	Truncated pancreas and the CHA arose from the SMA	Anterior sectionectomy (S5 and S8 resection) of the liver and partial resection of segment 3	NA	NA	36 months
2014 [11]	52	M	Hepatocellular carcinoma	None	Resection of liver segments 7 and 5	NA	NA	9 days
2013 [1]	74	M	Common bile duct carcinoma	Infiltrating the head of the pancreas	Pancreaticoduodenectomy	T3N1M0	NA	NA
2013 [1]	67	M	Adenocarcinoma of the bile duct	The CHA arose from the SMA	Pancreaticoduodenectomy	T1N0M0	NA	NA
2012 [12]	33	M	Adenocarcinoma of common bile duct	Infiltrating the head of pancreas	Cephalic pancreaticoduodenectomy	T3N1M0	NA	8 months

*F* female, *M* male, *CHA* common hepatic artery, *SMA* superior mesenteric artery, *NA* not available

Most often, even though CT or magnetic resonance imaging (MRI) easily identifies organ transposition, they do not clearly reveal whether it is a full or partial symmetry. Knowledge on the correct symmetry is very important before surgery to improve quality and accuracy of surgery [13]. In our case, we could not clearly locate the anatomical position of liver, SMV and colon. The greater size of the liver on the right created the impression there was no organ transposition. However, a careful examination of the portal system revealed transposition of the left branch “sagittal” and the right branch “anterior and posterior bifurcation”. Wrong judgment would have inevitably complicated liver lobe or partial liver resection. Normal SMV runs through the left side of the 2nd segments of the duodenum and behind the pancreas neck, whereas as for the patient, the SMV was still seen on the left side of the 2nd segments of the duodenum but distant to the pancreas. The splenic vein passed behind the 2nd segments of the duodenum and the pancreas to the spleen. Therefore, SMV was the only anatomical structure not exhibiting mirror symmetry, thus further complicating the resection of the duodenum and pancreatic head. The Kocher technique is applied for free SMV, and at the same time, because the SMV is located on the left side of duodenum rather than behind the neck of pancreas, the amputation of pancreatic uncinate process becomes relatively simple. Because the whole colon was still in the left abdominal cavity, it was only partially symmetrical, thus the normal transverse anatomy of the colon was lost. Therefore, accurate preoperative identification of the unique malformations provided invaluable information on how the horizontal segment of the duodenum, the lower edge of the pancreas and the transverse colon would be dissected.

The other more frequent anomalies in SIT include a relatively short pancreas, symmetric lobulation of the liver, biliary atresia, absence of the gallbladder, genitourinary anomalies and asplenia or polysplenia; transposition of blood vessels, nerves and lymphatics [14, 15]. The most visible abnormalities in the study patient were hepatic artery deformities and polysplenia. The hepatic artery exhibited Michels IX type malformation, where the CHA arises from SMA and this incidence is 4.5% [1], passes through the head of the pancreas and reversely replaced the gastroduodenal artery (GDA). After passing through the pancreas, it divides into left and right hepatic arteries before entering the liver. This presents two problems; Fist, the pancreatic tumor had spread 1 cm in to CHA that originated from SMA. If R0 tumor resection is considered, then common hepatic artery must also be resected and reconstructed. Second, since the proximal end of the severed common hepatic artery had retracted into the head of the pancreas, if a segment of hepatic artery was to be dissected from the pancreatic head and anastomosed with the splenic artery to complete the reconstruction of CHA blood flow, it would cut arterial blood supply to the pancreatic head. Accordingly, we performed resected the whole pancreas and duodenum. This was one of the most difficult and risky procedures, and precise judgment and pre-surgical design was very instrumental in successful operation.

The tumor displayed the following characteristics: (1) The gallbladder and pancreatic tumor were discrete, so it was not clear whether they were secondary or double primary tumors. (2) Pancreatic tumor had spread to the CHA. (3) Because the tumor was large, it was not clear whether the multiple organ invasions were primary tumors or metastases. Therefore, SIT could lead to wrong to erroneous late stage diagnosis of a tumor, in which surgery may be wrongly ruled out. However, accurate assessment of several scenarios informed our choice for surgery: (1) the cancer had not spread to main blood vessels (SMA, SMV, etc.), and CHA could still be partially removed and reconstructed. (2) R0 resection for stomach, intestine, pancreas, spleen and extrahepatic bile duct could be performed, (3) given that the tumor had not spread to the liver and its vasculature, there was no conflict between resectable mass volume and residual liver volume, (4) there were no obvious metastases in the peritoneum, omentum and mesangium and other distant organs. In the end, we achieved R0 resection for stomach, the common hepatic duct, jejunum and retroperitoneum. Meanwhile, pathological examination and immunohistochemical analysis of the cancer biopsies revealed that the gallbladder tumor had not spread to the pancreatic tail, which provided a very strong reference point for prognosis and subsequent chemotherapy regimens.

In conclusion, the complex SIT anatomy constraints tumor resection but is not a contraindication. In the case that the anatomy of the hepatobiliary-pancreatic region is not straightforward, the challenges of multicenter hepatobiliary and pancreatic tumor resection brought by SIT are not solvable for every surgeon, especially with vascular variants. Therefore, the surgical treatment of such patients requires detailed preoperative imaging to develop a reliable surgical resection plan, and careful exploration during the operation to achieve radical tumor resection, and the detailed operation process of this report provides a reference. Meanwhile, the final pathological examination is crucial for the diagnosis of tumor origin and the next therapeutic schedule for patients with multicenter tumors.

## Data Availability

All data generated or analyzed during this study are included in this published article. More information during the current study is available from the corresponding author on reasonable request.

## Abbreviations

SIT: Situs inversus totalis
CHA: Common hepatic artery
SMA: Superior mesenteric artery
CT: Computerized tomography
SMV: Superior mesenteric vein
MRI: Magnetic resonance imaging
GDA: Gastroduodenal artery
